# Relief from neuropathic pain by blocking of the platelet-activating factor–pain loop

**DOI:** 10.1096/fj.201601183R

**Published:** 2017-03-24

**Authors:** Hideo Shindou, Seiji Shiraishi, Suzumi M. Tokuoka, Yoshikazu Takahashi, Takeshi Harayama, Takaya Abe, Kana Bando, Kanako Miyano, Yoshihiro Kita, Yasuhito Uezono, Takao Shimizu

**Affiliations:** * Department of Lipid Signaling, National Center for Global Health and Medicine, Tokyo, Japan;; † Agency for Research and Medical Development (AMED), Tokyo Japan;; ‡ Department of Lipid Science, Graduate School of Medicine, The University of Tokyo, Tokyo, Japan;; ¶ Department of Lipidomics, Graduate School of Medicine, The University of Tokyo, Tokyo, Japan; and; ** Life Sciences Core Facility, Graduate School of Medicine, The University of Tokyo, Tokyo, Japan;; § Division of Cancer Pathophysiology, National Cancer Center Research Institute, Tokyo, Japan;; ‖ Genetic Engineering Team, RIKEN Center for Life Science Technologies, Kobe, Japan; and; # Animal Resource Development Unit, RIKEN Center for Life Science Technologies, Kobe, Japan; and; †† Division of Supportive Care Research, Exploratory Oncology Research and Clinical Trial Center, National Cancer Center, Tokyo, Japan

**Keywords:** PAF, lysophosphatidylcholine acyltransferase 2, LPCAT2, analgesic drug, feedback loop

## Abstract

Neuropathic pain resulting from peripheral neuronal damage is largely resistant to treatment with currently available analgesic drugs. Recently, ATP, lysophosphatidic acid, and platelet-activating factor (PAF) have been reported to play important inductive roles in neuropathic pain. In the present study, we found that pain-like behaviors resulting from partial sciatic nerve ligation (PSL) were largely attenuated by deficiency of lysophosphatidylcholine acyltransferase (LPCAT)2, which is one of the PAF biosynthetic enzymes. By contrast, deficiency of the other PAF biosynthetic enzyme, LPCAT1, did not ameliorate neuropathic pain. With regard to the mechanism of the observed effects, LPCAT2 was detected in wild-type spinal cord microglia, and the absence of LPCAT2 expression precluded spinal PAF expression in LPCAT2-knockout mice. Furthermore, ATP-stimulated PAF biosynthesis in macrophages was decreased by pretreatment with the PAF receptor antagonist ABT-491, indicating the existence of a positive feedback loop of PAF biosynthesis, which we designated the PAF–pain loop. In conclusion, LPCAT2 is a novel therapeutic target for newly categorized analgesic drugs; in addition, our data call for the re-evaluation of the clinical utility of PAF receptor antagonists.—Shindou, H., Shiraishi, S., Tokuoka, S. M., Takahashi Y., Harayama, T., Abe, T., Bando, K., Miyano, K., Kita, Y., Uezono, Y., Shimizu, T. Relief from neuropathic pain by blocking of the platelet-activating factor–pain loop.

Neuropathic pain is a chronic pain condition that can be initiated by injury or perturbation of the peripheral nervous system or the CNS. Current therapy for neuropathic pain is limited, and analgesic drugs, such as nonsteroidal anti-inflammatory drugs (NSAIDs) and opioids, are ineffectual. The development of effective analgesics for the management of neuropathic pain is an important unmet medical need. Several biomolecules have been implicated in neuropathic pain, including ATP, lysophosphatidic acid, and platelet-activating factor (PAF) (1–3). Nerve injury is known to induce the expression of the P2X4 and P2Y12 receptors, brain-derived neurotrophic factor, and PAF-related genes in the spinal cord dorsal horn in association with neuropathic pain (4–8). Recently, Masuda *et al.* (9) reported that microglia are also activated by ATP released from dorsal horn neurons *via* a vesicular nucleotide transporter.

In the present study, we focused on the role of PAF signaling in neuropathic pain. PAF is a potent lipid mediator that is biosynthesized in response to extracellular stimuli by lyso-PAF acetyltransferase using lyso-PAF and acetyl-CoA as substrates (10, 11). To date, 2 types of lyso-PAF acetyltransferase enzymes have been identified in our group: lysophosphatidylcholine acyltransferase (LPCAT)1 and LPCAT2 (11–13). We previously reported that in macrophages, LPCAT2 but not LPCAT1 is phosphorylated in response to 30 s stimulation with PAF or ATP, and 30 min stimulation with LPS, resulting in enhanced lyso-PAF acetyltransferase activity (11, 14, 15). Moreover, LPS stimulation for a longer period induces LPCAT2 expression in macrophages (11, 12). Thus, the relationship between LPCAT1 and -2 is similar to that of housekeeping cyclooxygenase (*Cox*)*-1* and inducible *Cox-2* (16). Recently, we screened 170,000 compounds and identified a specific inhibitor of LPCAT2, TSI-01 (17). TSI-01 was found to suppress PAF production in macrophages.

To further elucidate the role of PAF in neuropathic pain in the present study, we constructed an LPCAT2-knockout (KO) mouse line (Supplemental Fig. S1). We found that PAF was almost completely absent in the spinal cord, macrophages, and several other tissues of LPCAT2-KO mice. Moreover, neuropathic pain was attenuated in the absence of LPCAT2, but not LPCAT1 in a model of partial sciatic ligation (PSL). The suppression of PAF biosynthesis by pretreatment with a PAF receptor (PAFR) antagonist, ABT-491, suggests that a positive feedback loop of PAF biosynthesis (the PAF–pain loop) may function to exacerbate neuropathic pain. Our findings present a new concept of analgesic drug development for neuropathic pain through the inhibition of the PAF biosynthetic enzyme LPCAT2 and re-evaluation of PAFR antagonists.

## MATERIALS AND METHODS

### Animals

All animal experiments were approved by and performed in accordance with the guidelines of the Animal Research Committee of National Center for Global Health and Medicine (12053, 13009, 14045, 15037, and 16062), the Animal Experimentation Committee of the University of Tokyo (P08-042 and P08-046), and the Institutional Animal Care and Use Committee of RIKEN, Kobe Branch (AH13-03).

### Generation of LPCAT2-KO mice

LPCAT2-floxed mice (CDB0649K: *http://www2.clst.riken.jp/arg/mutant%20mice%20list.html*) were generated as described (*http://www2.clst.riken.jp/arg/methods.html*), using the HK3i embryonic stem cells from the C57Bl/6N strain (18). To generate a targeting vector, genomic fragments of the *LPCAT1* locus were obtained from the RP23-58E22 BAC clone (BACPAC Resources, Oakland, CA, USA). A 534-bp gene region containing exon 3 of *LPCAT2* was flanked with loxP sites (Supplemental Fig. S1). Targeted embryonic stem cell clones were microinjected into ICR 8-cell stage mouse embryos, and the injected embryos were transferred into pseudopregnant ICR female mice. The resultant chimeric mice were bred with C57BL/6 mice, and heterozygous offspring were identified by PCR. Exon 3 of LPCAT2 was removed by breeding heterozygous offspring with telencephalin-Cre mice (19, 20) (backcrossed at least 6 times on a C57Bl/6N background), in which Cre-mediated recombination takes place throughout the body at the postimplantation stage. The Cre allele was removed by mating with C57Bl/6N mice to obtain LPCAT2 heterozygous mice, which were used for establishment of LPCAT2 homozygous mice. For genotyping, DNA was extracted from tail tip samples and subjected to PCR with Ex Taq HS DNA polymerase (Takara Bio, Shiga, Japan). Primers were designed to amplify 772-bp (wild-type; WT) and 336-bp (LPCAT2-KO) fragments: forward, 5′-CTCAAGACAGGACCTTGGAGTCA-3′; reverse, 5′-ACTGGCTGGAGATATCATTCGGT-3′.

### PSL model

PSL was performed as previously reported (21). In brief, 8-wk-old male WT, LPCAT2-KO, or LPCAT1-KO (22) mice were anesthetized with isoflurane and partial ligation of the sciatic nerve was performed by tying off the distal one-third to one-half of the sciatic nerve connected to the spinal cord (at vertebrae L3–L5) according to the procedure described in rats by Seltzer *et al*. (23) and adapted for mice by Malmberg and Basbaum (24). In sham-surgery mice, the nerve was exposed by using the same procedure, but not ligated. Two weeks after surgery, allodynia-like behavior of the PSL- and sham-surgery mice was evaluated by the paintbrush and von Frey filament tests (Muromachi Kikai, Tokyo, Japan). Allodynia-like behavior was assessed by stroking the injured leg with a paintbrush (allodynia score) and by measuring the paw withdrawal threshold in response to probing with a series of calibrated fine filaments [Tactile test (Aesthesio); Muromachi Kikai] (25). The allodynia score was ranked as described by Minami *et al*. (26): 0, no response; 1, mild squeaking with attempts to move away from the paintbrush; and 2, vigorous squeaking, biting the paintbrush, and strong efforts to escape the paintbrush.

### Isolation and stimulation of mouse peritoneal macrophages

Mouse peritoneal macrophages were isolated as described elsewhere (27). In brief, 3 d after intraperitoneal injection of 2 ml 4% thioglycollate, peritoneal macrophages were harvested from the peritoneal cavity with ice-cold PBS. Cells (3.2 × 10^6^ cells/6-cm dish and 3.2 × 10^5^ cells/well on a 12-well plate) were cultured in RPMI-1640 medium supplemented with 10% fetal bovine serum at 37°C in a humidified atmosphere of 5% CO_2_. After incubation for 2 h, the cells were stimulated with 100 ng/ml LPS for 30 min or 18 h. For the preparation of cell extracts, the cells were scraped with 600 µl of an ice-cold buffer containing 20 mM Tris-HCl, (pH 7.4), 300 mM sucrose, 1 mM sodium orthovanadate, and 1× EDTA-free proteinase inhibitor cocktail Complete (Roche, Basel, Switzerland), and the collected cells were sonicated on ice twice for 30 s each. Intact cells, cellular debris, and mitochondria were removed by centrifugation at 9000 *g* for 10 min at 4°C. The resultant supernatants were centrifuged at 100,000 *g* for 1 h at 4°C, and pellets were finally resuspended in buffer containing 20 mM Tris-HCl (pH 7.4), 300 mM sucrose, and 1 mM sodium orthovanadate. The microsomal protein concentration of each sample was measured by the Bradford method (Bio-Rad, Hercules, CA, USA), with bovine serum albumin (fraction V, fatty acid-free; Sigma-Aldrich, St. Louis, MO, USA) as a standard. For the measurement of PAF, cells were stimulated with 5 µM Ca ionophore, A23187 (Enzo Life Sciences, Farmingdale, NY, USA) for 5 min in HBSS buffer or RPMI-1640 medium with 10% fetal bovine serum. The reaction was stopped by the addition of equivalent volumes of methanol and washed by methanol again with spiked internal standards. The extraction and purification of PAF are described later.

### Assessment of the PAF biosynthesis feedback loop

Peritoneal macrophages were pretreated with or without PAFR antagonists ABT-491 100 nM (28) (Sigma-Aldrich) and WEB2086 30 µM (BioTechne, Minneapolis, MN, USA) for 10 min and subsequently stimulated with 1 mM ATP (Sigma-Aldrich) for the indicated times. The reaction was stopped by the addition of methanol after the aspiration of cell culture medium.

### Western blot analysis

Protein samples were resolved on 10% SDS-polyacrylamide gels and electrophoretically transferred to nitrocellulose membranes (GE Healthcare, Tokyo, Japan) using a Trans-Blot transfer cell (Bio-Rad). Membranes were blocked for more than 16 h with 5% skim milk (BD Biosciences, Franklin Lakes, NJ, USA). Anti-LPCAT2 (1:1000), anti-phospho-LPCAT2 (1:1000), and anti-calnexin (1:100) (BD Biosciences) antibodies were used as the primary antibodies. Anti-LPCAT2 and anti-phospho-LPCAT2 antibodies were available from another study (13). Horseradish peroxidase–conjugated secondary antibodies (1:2000; GE Healthcare) were used. ECL select Western blot detection system (GE Healthcare) was used for chemiluminescence, and detected using ImageQuant LAS500 (GE Healthcare).

### Lyso-PAF acetyltransferase (PAF biosynthesis) and acyltransferase (phosphatidylcholine biosynthesis) assays

Microsomal protein fractions (1 µg) were added to reaction mixtures containing 100 mM Tris-HCl (pH 7.4), 1 mM CaCl_2_, 0.015% Tween-20, 0.5 mM EDTA, and 2 substrates. For acetyltransferase activity, the substrates were 1 mM acetyl-CoA (Wako, Osaka, Japan) and 5 µM lyso-PAF-d4 (Cayman Chemical, Ann Arbor, MI, USA); for acyltransferase activity, the substrates were 25 µM arachidonoyl-CoA (Avanti Polar Lipid, Alabaster, AL, USA) and 5 µM lyso-PAF-d4. After incubation at 37°C for 10 min, the reactions were stopped by the addition of 300 µl methanol containing 17:0 LPC (Cayman) or dimyristoyl-phosphatidylcholine (PC; Cayman Chemical) as internal standards. Deuterium-labeled products were analyzed by liquid chromatography-tandem mass spectrometry (LC-MS/MS; Waters, Milford, MA, USA, and Thermo Fisher Scientific, Waltham, MA, USA) (15). Products were separated on an Acquity ultra performance liquid chromatography BEH C8 column (1.7 µm, 2.1 × 30 mm; Waters) using a linear gradient of solvent B (acetonitrile; Wako) over solvent A (20 mM NH_4_HCO_3_/water; Wako) with an Acquity Ultra-r/cPerformance Liquid Chromatography (UPLC) system (Waters). The flow rate was 800 µl/min. The gradient started at 55% solvent B and was linearly increased to 95% solvent B over 4.5 min and maintained for 1.5 min. Detection was performed on a TSQ Vantage triple-stage quadrupole mass spectrometer (Thermo Fisher Scientific) by selected reaction monitoring (SRM). Transitions were [M+H]+ → 184.1 for PAF and PC in positive ion mode electrospray ionization. Product signals were compared to calibration curves of nonlabeled standards for quantification.

### Measurement of PC from tissues

Frozen tissues (20–110 mg) were pulverized and extracted in 0.8 ml methanol for 60 min at 4°C. The tissue suspensions were centrifuged at 15,000 *g* for 10 min at 4°C, and the collected supernatant was used for the measurement of phospholipids. The methanol extracts [2.6–5.6 mg tissue/ml (liver), 3.1–4.6 mg tissue/ml (spleen), 6.1–13.5 mg tissue/ml (kidney), 8.5–11.3 mg tissue/ml (brain), 3.3–7.3 mg tissue/ml (thymus), and 2.3–4.1 mg tissue/ml (colon)] were used for the measurement of phospholipids. From macrophages and spinal cord, lipids were extracted with a solid-phase method with an Oasis HLB cartridge (Waters) (29, 30). After solid-phase extraction, samples were diluted with methanol at a concentration of 1.6 × 10^6^ cells/ml (macrophages) and 5–7.5 mg tissue/ml (spinal cord), and used for the measurement of phospholipids. Liquid chromatography-selected reaction monitoring-mass spectrometry was performed with a Nexera Ultra High-Performance Liquid Chromatography (UHPLC) system and a triple quadrupole mass spectrometer (LCMS-8040; Shimadzu, Kyoto, Japan). An Acquity UPLC BEH C8 column (1.7 μm, 2.1 × 100 mm; Waters) was used with the following ternary mobile phase compositions: 5 mM NH_4_HCO_3_/water (mobile phase A), acetonitrile (mobile phase B), and isopropanol (mobile phase C). The pump gradient [time (%A/%B/%C)] was programmed as follows: 0 min (75/20/5), 20 min (20/75/5), 40 min (20/5/75), 45 min (5/5/90), 50 min (5/5/90), and 55 min (75/20/5). The flow rate was 0.35 ml/min, and column temperature was 47°C. Injection volume was 5 μl. SRM analysis was performed with the transitions [M+H]+ →184 for PC species possessing 12–24 carbon fatty acyl chains in *sn*-1 and *sn*-2. Peak areas of individual species were normalized against the sum of all signals for each sample.

### Measurement of PAF

For the quantification of PAF, an internal standard PAF-d4 was added to methanol extracts, and samples were purified with solid-phase extraction with an Oasis HLB cartridge (Waters) (29, 30). PAF was quantified by triple quadrupole mass spectrometers LCMS-8050 and -8080 (Shimadzu). Separation was performed on a Kinetex C8 column (2.6 μm, 2.1 × 150 mm; Phenomenex, Torrance, CA, USA) with a binary mobile phase of the following compositions: 0.1% formic acid/water (mobile phase A) and acetonitrile (mobile phase B). Pump gradient [time (%A/%B)] was programmed as follows: 0 min (90/10), 5 min (75/25), 10 min (65/35), 20 min (25/75), 20.1 min (5/95), 28 min (5/95), 28.1 min (90/10), and 30 min (90/10). Flow rate was 0.4 ml/min, and column temperature was 40°C. SRM transitions were 568.4→59.1 for PAF and 572.4→59.1 for PAF-d4, in negative ion mode. For quantification, signals were compared to standard curves (29, 30).

### Histologic analysis

Spinal cords were fixed in a solution of 4% paraformaldehyde (Wako) for 24 h. After fixation, tissues were embedded in paraffin and sections were cut by the BoZo Research Center Inc. (Tokyo, Japan). Next, immunohistochemistry (IHC) was performed by GenoStaff (Tokyo, Japan). For the first IHC, tissue sections were incubated with anti-LPCAT2 antibody at 4°C overnight. The sections were incubated with biotin-conjugated goat anti-rabbit IgG (Dako, Tokyo, Japan) for 30 min at room temperature, followed by the addition of alkaline phosphatase–conjugated streptavidin (Nichirei, Tokyo, Japan) for 10 min. Color detection was performed with nitroblue-tetrazolium/5-bromo-4-chloro-3-indolyl phosphate solution (NBT/BCIP; Sigma-Aldrich) and the microscopic examinations were performed at GenoStaff. For the second IHC, tissue sections were incubated with anti-ionized calcium-binding adapter molecule 1 (Iba1) antibody (Wako) at 4°C overnight. Then, sections were incubated with biotin-conjugated goat anti-rabbit IgG (Dako, Tokyo, Japan) for 30 min at room temperature followed by the addition of peroxidase-conjugated streptavidin (Nichirei) for 5 min. Peroxidase activity was visualized with diaminobenzidine. Stained sections were then mounted with CC/Mount (Diagnostic BioSystems, Pleasanton, CA, USA) and microscopic examinations were performed at GenoStaff (Tokyo, Japan).

### Statistical analysis

All statistical calculations were performed using Prism 5 and 6 software (GraphPad Software, La Jolla, CA, USA).

## RESULTS

### Suppression of PAF biosynthesis in LPCAT2-KO mice

**Figure 1.**
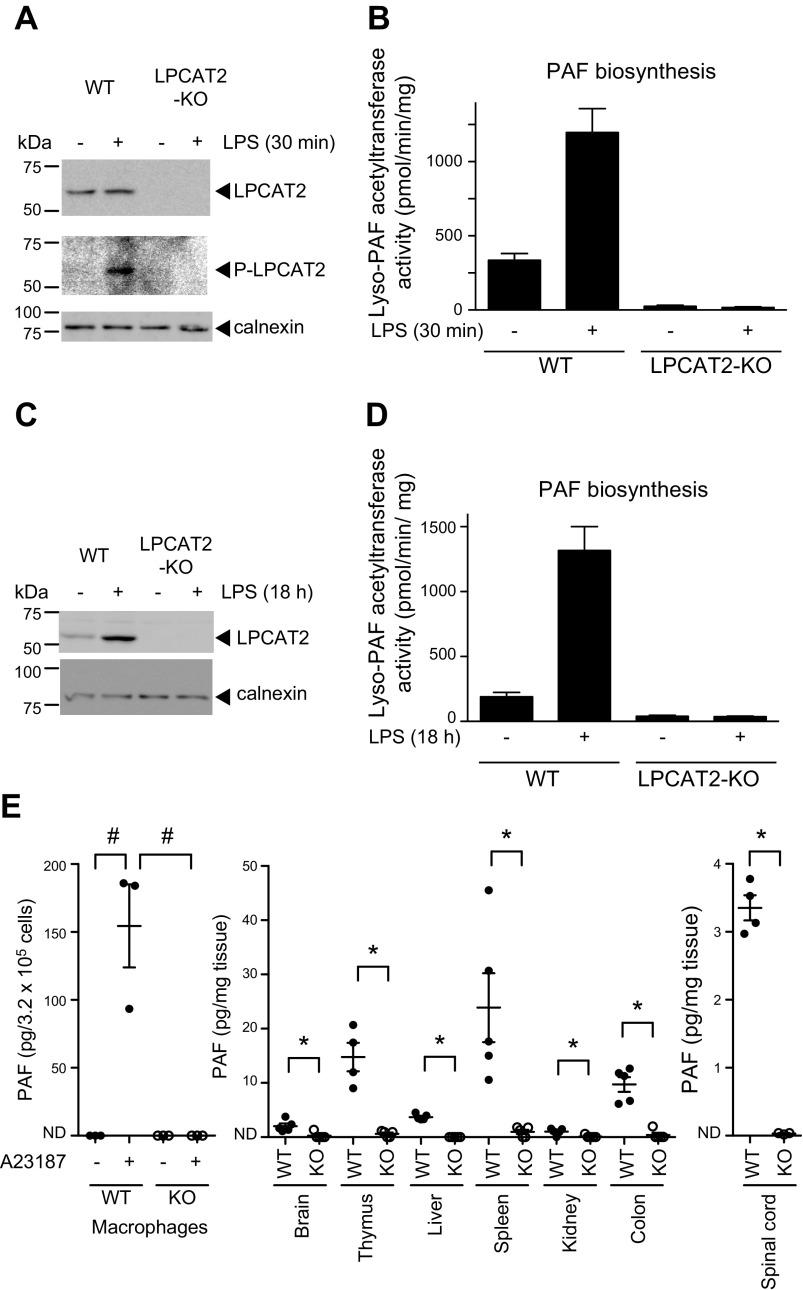
LPCAT2 deficiency decreased PAF production. *A*–*D*) Peritoneal macrophages were stimulated with LPS for 30 min (*A*, *B*) or 18 h (*C*, *D*) (*n =* 3). *A*, *B*) Phosphorylation (*A*) and activation (*B*; PAF biosynthetic activity) of LPCAT2 were detected in WT, but not in LPCAT2-KO, cells. *C*, *D*) Increased protein expression (*C*) and LPCAT2 activity (*D*; PAF biosynthetic activity) were also observed in cells from WT but not LPCAT2-KO mice. Calnexin was used as an internal control. *E*) PAF production was examined in peritoneal macrophages stimulated with 5 µM A23187 for 5 min (*n =* 3) and in several tissues, including brain (*n =* 5), thymus (WT; *n =* 4, KO; *n =* 5), liver (*n =* 5), spleen (*n =* 5), kidney (*n =* 5), colon (*n =* 5), and spinal cord (WT; *n =* 4, KO; *n =* 3). PAF was almost undetectable in LPCAT2-KO tissues. All data are expressed as means ± se. ^#^*P* < 0.05, 1-way ANOVA with Kruskal-Wallis and Dunn's multiple-comparison tests; *P < 0.05, Mann-Whitney *U* test.

PAF is rapidly synthesized by LPCAT2 in cells that respond to inflammatory stimulation. To investigate whether LPCAT2 is the only enzyme producing PAF under inflammatory conditions, we analyzed LPCAT2 activities in peritoneal macrophages collected from LPCAT2-KO mice. In WT cells, but not LPCAT2-KO cells, LPS stimulation for 30 min and 18 h enhanced PAF biosynthetic activity (lyso-PAF acetyltransferase), indicating its phosphorylation and upregulation, respectively (**Fig. 1*A*–*D***). Lyso-PAF acyltransferase activity (biosynthesis of PC with arachidonic acid), which is another characteristic of LPCAT2, was decreased but not absent in LPCAT2-KO cells (Supplemental Fig. S2), possibly because of the activity of other acyltransferases such as LPCAT3.

In LPCAT2-KO mice, PAF production was not detected in macrophages after stimulation with the Ca ionophore A23187 or in tissues, including the spinal cord, under steady-state conditions (Fig. 1*E*). The abundance of other PCs were similar between WT and LPCAT2-KO mice (Supplemental Fig. S3). Based on these results, LPCAT2 is indeed a sole regulatory PAF biosynthetic enzyme in macrophages.

### Reduction of neuropathic pain–induced allodynia by LPCAT2 gene disruption

**Figure 2.**
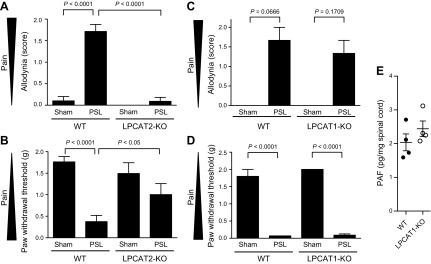
PSL-induced pain model. *A*–*D*) PSL was performed in LPCAT2-KO (*A*, *B*) and LPCAT1-KO (*C*, *D*) mice, and the resultant pain level was evaluated by using the paintbrush (*A*, *C*) and von Frey (*B*, *D*) tests. Sham-test mice in *C* did not show any pain. Values are means ± se: WT/sham test (*n =* 11), WT/PSL (*n =* 14), LPCAT2-KO/sham test (*n =* 10), and LPCAT2-KO/PSL (*n =* 11)(*A*, *B*); WT (*n =* 3) and LPCAT1-KO (*n =* 3) (*C*, *D*). *P* values: 1-way ANOVA with Kruskal-Wallis and Dunn’s multiple-comparison tests (*A*, *C*); 2-way ANOVA with Bonferroni test (*B*, *D*). *E*) PAF level of spinal cord from LPCAT1-KO was similar to that of WT. Values are means ± se of results in 4 independent experiments.

We next used a model of PSL-induced neuropathic pain in WT and LPCAT2-KO mice. Sham- and PSL-surgery mice were evaluated for allodynia-like behavior based on the paintbrush and von Frey filament tests (25). PSL increased pain behaviors in WT mice, but not in LPCAT2-KO mice (**Fig. 2*A*, *B***). The score of sham-surgery LPCAT2-KO mice was almost the same as that of WT mice. Alternatively, deficiency of LPCAT1, which is the other PAF biosynthetic enzyme with high homology to LPCAT2, did not ameliorate pain behavior (Fig. 2*C*, *D*). PAF levels in spinal cord were very low in LPCAT2-KO compared with WT mice (Fig. 1*E*), but not changed in LPCAT1-KO mice (Fig. 2*E*).

### Expression of LPCAT2 in microglia in spinal cord dorsal horn

**Figure 3.**
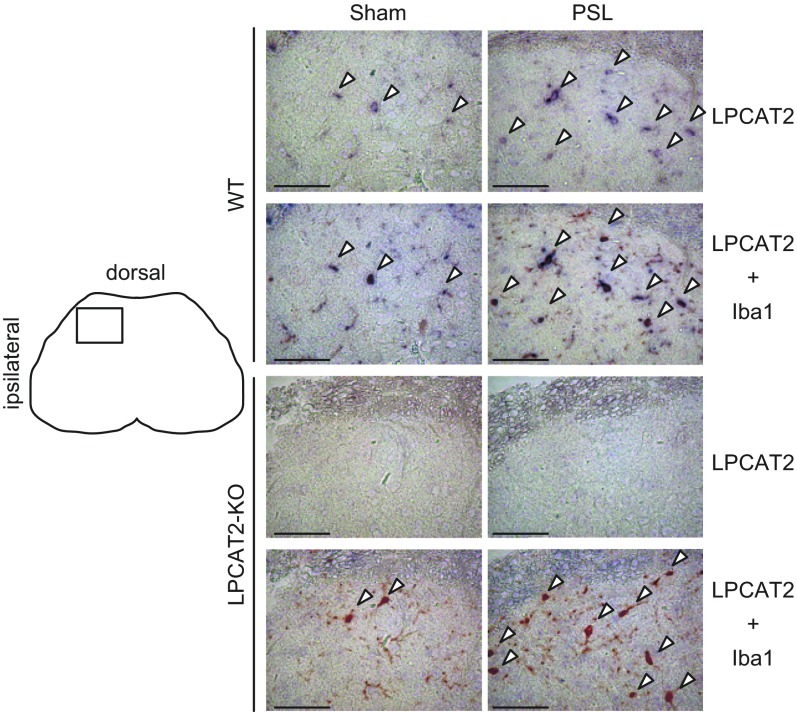
Expression of LPCAT2 in microglia of the spinal cord dorsal horn. Expression of LPCAT2 and microglia were detected with anti-LPCAT2 antibody/NBT-BCIP (purple) and anti-Iba1 antibody/diaminobenzidine (brown), respectively. Arrowheads: LPCAT2 and Iba 1 localization. Four independent experiments were performed with similar results. Scale bars, 50 µm.

Next, we examined LPCAT2 expression in the spinal cord. Numbers of Iba1 positive microglia (referred to as “microglia” in this article) in the lumbar (L3–L5) spinal cord dorsal horn were increased after PSL surgery, and moreover, LPCAT2 was detected in these microglia (**Fig. 3**). In LPCAT2-KO mice, microglia increase was also observed, whereas LPCAT2 was not detected in spinal cord. Taken together, these data suggest that LPCAT2 expression and PAF production in microglia of the spinal cord dorsal horn play a role in neuropathic pain after nerve injury. From the results, PAF produced by LPCAT2 is important in the cause and retention of neuropathic pain.

### Existence of the positive-feedback loop of PAF biosynthesis

**Figure 4.**
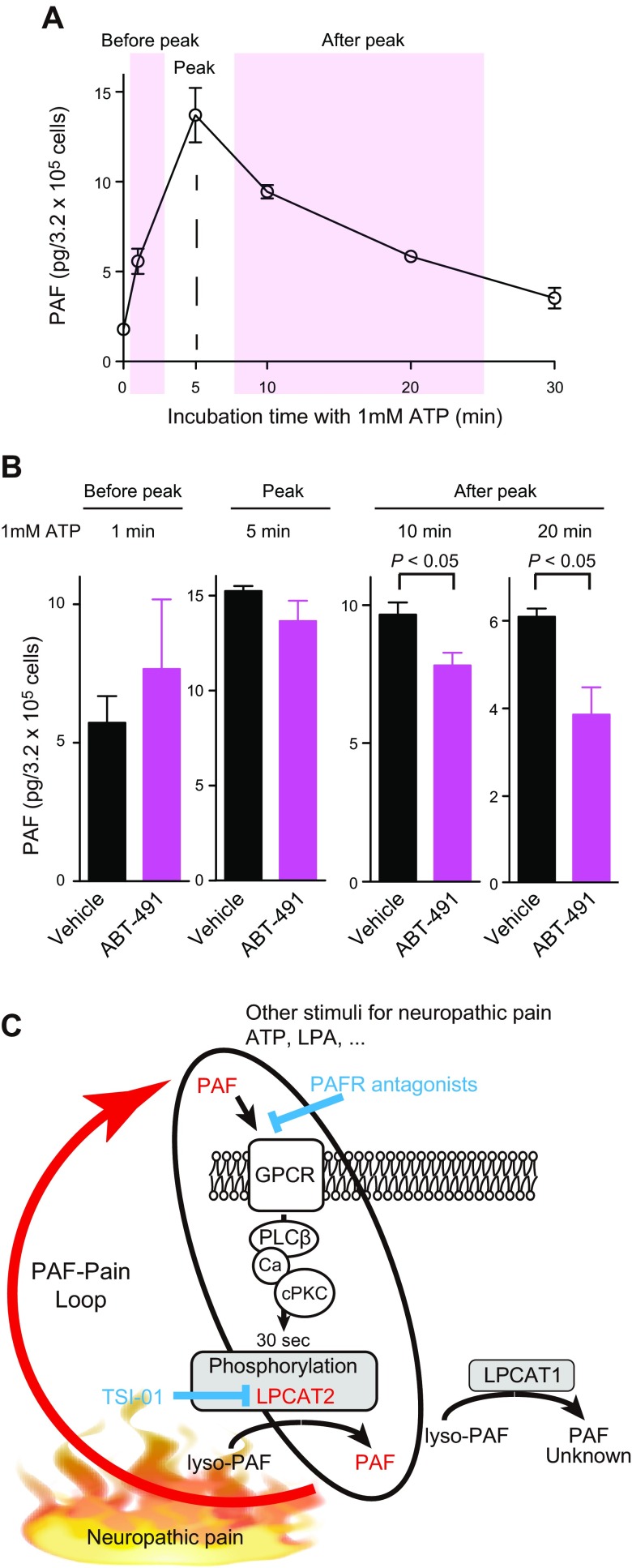
PAF–pain loop. *A*) Peritoneal macrophages were stimulated with 1 mM ATP, and PAF synthesis was measured. PAF production peaked at 5 min after stimulation. Values are means ± se of results in 4 experiments. *B*) Pretreatment with PAFR antagonist ABT-491 (100 nM) for 10 min decreased PAF production after peak (late phase), but not before. Vehicle control data (*n =* 3) are the same as depicted in *A*. Data are expressed as means ± se of results in 3 independent experiments. *P* < 0.05, unpaired *t* test. *C*) Schematic representation of the proposed PAF–pain loop. PAF and/or ATP stimulate cells to enhance PAF production. Next, PAF in turn activates nearby cells and further increases PAF production in a positive feedback loop. Neuropathic pain may be exacerbated and perpetuated by the PAF–pain loop. TSI-01 is a specific inhibitor of LPCAT2.

In macrophages, PAF and ATP stimulation are known to induce LPCAT2 phosphorylation to produce PAF (15). Thus, we investigated whether PAF produced in response to ATP can coordinately increase PAF biosynthesis by way of a positive-feedback loop. In peritoneal macrophages, the peak PAF level was observed 5 min after ATP stimulation (**Fig. 4*A***). Next, cells were pretreated with the PAFR antagonist ABT-491 (28) and subsequently subject to ATP stimulation; PAF biosynthesis was not altered at 1 or 5 min after stimulation, indicating that PAF biosynthesis was initiated by ATP rather than loop activation of PAFRs (before peak). However, at 10 and 20 min after ATP stimulation, PAF production was suppressed by ABT-491 pretreatment (Fig. 4*B*), indicating that the secondary phase of PAF biosynthesis in response to ATP stimulation is dependent on PAFR activation (after peak). Pretreatment with an additional PAFR antagonist WEB2086 also decreased ATP-stimulated PAF production at the late phase (Supplemental Fig. S5). The data suggest the presence of a positive feedback loop of PAF biosynthesis in cells that may be involved in sustaining neuropathic pain (Fig. 4*C*).

## DISCUSSION

Overall, this study identified LPCAT2 but not LPCAT1 as a key molecule for PAF production and neuropathic pain evaluated in a PSL model. Notably, PAF production was not observed in the LPCAT2-KO spinal cord. In addition, since the later phases of PAF production by ATP stimulation were suppressed in the presence of ABT-491 (PAFR antagonist), PAF is thought to be biosynthesized in the positive-feedback loop, which may induce neuropathic pain by constructing the PAF–pain loop (Fig. 4*C*). ATP is also known to promote neuropathic pain by eliciting the hyperexcitability of dorsal horn neurons (1, 5). Combined with previous reports, the present study suggests that the PAF–pain loop is a key component underlying neuropathic pain (Fig. 4*C*).

To date, 2 PAF biosynthetic enzymes have been identified: the constitutive type LPCAT1 and the inducible type LPCAT2 (11–13). The relationship between LPCAT1 and -2 is therefore similar to that between Cox-1 and -2 (16). Because the exact biologic roles of PAF produced by LPCAT1 are currently unclear and require further elucidation (31), and, given that LPCAT1 deficiency produces respiratory damage and visual dysfunction in mice (22, 32), specific inhibition of LPCAT2 appears to be a safer therapeutic strategy than PAFR inhibition. However, the reevaluation of PAFR antagonists in the context of neuropathic pain is also important and may represent a faster avenue to clinical application, given that many pharmaceutical companies have already developed PAFR antagonists as anti-inflammatory drugs and have screened toxicity in phase I trials, but have not marketed them because of a lack of efficacy (33).

As mentioned, an important finding of this study was the presence of a positive feedback loop for PAF biosynthesis in macrophages. From our results, we speculated that this loop underlies or exacerbates sustained neuropathic pain as the PAF–pain loop (Fig. 4*C*). The future use of PAFR-KO mice (in addition to the use of pharmacological agents) will assist in understanding this loop. Moreover, given that intrathecal injection of a PAFR antagonist attenuates neuropathic pain (21) and PAF-induced allodynia (34), the spinal cord is an important candidate area for functional study of the PAF–pain loop. Finally, LPCAT2 and PAFR were expressed in microglia of the spinal cord dorsal horn (7), but the roles of other cells in the production and action of PAF should be investigated in the context of the PAF–pain loop. We also observed the induction of microglia expressing LPCAT2 in the ventral horn proximal to motor neurons (Supplemental Fig. S4), although the relationship between the increased number of microglia in the ventral horn and neuropathic pain is unknown. Furthermore, in this study, changes in PAF levels in spinal cord of WT mice that underwent PSL was not detected (data not shown). Detection of PAF changes may be difficult in the conditions set up by current approaches, since PAF is immediately catabolized by PAF acetylhydrolases (10, 35). To detect possible changes in PAF levels, we need to optimize spinal cord collection, both timing after the PSL operation and correct selection of the area. Basal levels of PAF were detected in spinal cords from WT and LPCAT1-KO mice, but not LPCAT2-KO mice. Effects of steady-state levels of PAF on neuropathic pain also are not understood. To overcome these problems and clarify the PAF–pain loop mechanisms spatiotemporally, refinement of LC-MS and imaging MS are needed in the future.

Neuropathic pain is considered to be resistant to NSAIDs, which inhibit COX enzymes and decrease the production of prostanoids (16, 36). The enzymatic products of LPCAT2 are not only PAF, but also PC with polyunsaturated fatty acids, including arachidonic acid (11, 12, 22). Although polyunsaturated fatty acids from PC also serve as precursors of lipid mediators (37, 38), we did not observe changes in PC compositions in the spinal cord or other tissues from LPCAT2-KO mice.

In summary, the present study uncovered the PAF–pain loop as an important potential biochemical mechanism of neuropathic pain. Combined with current knowledge regarding other pronociceptive molecules related to neuropathic pain, the PAF–pain loop could be a novel therapeutic target. Neuropathic pain fails to respond to the ordinary medication; thus, we need to develop effective analgesic drugs for the relief of neuropathic pain. Following NSAIDs and opioids, LPCAT2 inhibitors and PAFR antagonists may be candidates for development of novel drugs and facilitate improved clinical management for the relief of neuropathic pain.

## Abbreviations

BCIP: 5-bromo-4-chloro-3-indolyl phosphate
Cox: cyclooxygenase
IHC: immunohistochemistry
KO: knockout
LPCAT: lysophosphatidylcholine acyltransferase
NBT: nitroblue tetrazolium
NSAID: nonsteroidal anti-inflammatory drug
PAF: platelet-activating factor
PAFR: PAF receptor
PC: phosphatidylcholine
PSL: partial sciatic ligation
SRM: selected reaction monitoring
UPLC: ultra-performance liquid chromatography
WT: wild type
